# Employing Engineered Enolase Promoter for Efficient Expression of *Thermomyces lanuginosus* Lipase in *Yarrowia lipolytica* via a Self-Excisable Vector

**DOI:** 10.3390/ijms24010719

**Published:** 2022-12-31

**Authors:** Liangcheng Jiao, Wenjuan Li, Yunchong Li, Qinghua Zhou, Mengqin Zhu, Guowei Zhao, Houjin Zhang, Yunjun Yan

**Affiliations:** Key Laboratory of Molecular Biophysics of the Ministry of Education, College of Life Science and Technology, Huazhong University of Science and Technology, Wuhan 430074, China

**Keywords:** *Yarrowia lipolytica*, enolase promoter, markerless integration, *Thermomyces lanuginosus* lipase, heterologous overexpression

## Abstract

*Yarrowia lipolytica* is progressively being employed as a workhouse for recombinant protein expression. Here, we expanded the molecular toolbox by engineering the enolase promoter (pENO) and developed a new self-excisable vector, and based on this, a combined strategy was employed to enhance the expression of *Thermomyces lanuginosus* lipase (TLL) in *Y. lipolytica*. The strength of 11 truncated enolase promoters of different length was first identified using eGFP as a reporter. Seven of the truncated promoters were selected to examine their ability for driving TLL expression. Then, a series of enolase promoters with higher activities were developed by upstream fusing of different copies of UAS1B, and the recombinant strain Po1f/hp16e_100_-tll harboring the optimal promoter hp16e_100_ obtained a TLL activity of 447 U/mL. Additionally, a new self-excisable vector was developed based on a Cre/*lox*P recombination system, which achieved efficient markerless integration in *Y. lipolytica*. Subsequently, strains harboring one to four copies of the *tll* gene were constructed using this tool, with the three-copy strain Po1f/3tll showing the highest activity of 579 U/mL. The activity of Po1f/3tll was then increased to 720 U/mL by optimizing the shaking flask fermentation parameters. Moreover, the folding-related proteins Hac1, Pdi, and Kar2 were employed to further enhance TLL expression, and the TLL activity of the optimal recombinant strain Po1f/3tll-hac1-pdi-kar2 reached 1197 U/mL. By using this combined strategy, TLL activity was enhanced by approximately 39.9-fold compared to the initial strain. Thus, the new vector and the combined strategy could be a useful tool to engineer *Y. lipolytica* for high-level expression of heterologous protein.

## 1. Introduction

With the rapid growth of demand for recombinant proteins in food, bioenergy, pharmaceutical, and other industrial fields, improving the expression efficiency of target genes has become an important goal. Many proteins are expressed using the yeast-based system because of its ease of genetic manipulation and ability to perform post-translational modifications [1,2]. Besides the well-known *Saccharomyces cerevisiae*, other yeast species are also commonly used as platforms for the expression of heterologous proteins, including *Pichia pastoris*, *Kluyveromyces lactis*, *Schizosaccharomyces pombe*, *Hansanula polymorpha*, and *Yarrowia lipolytica* [2,3,4,5]. Among them, the dimorphic yeast *Y. lipolytica* is a non-conventional and non-pathogenic organism that has been classified as “generally recognized as safe” (GRAS) by the Food and Drug Administration [6]. It can grow on a variety of carbon source substrates, such as glucose, glycerol, alkanes, and fatty acids, and it has the innate ability to secrete proteases, lipases, and esterases [5,7]. Based on these excellent characteristics, *Y. lipolytica* has been employed for the heterologous expression of over 130 recombinant proteins [5].

To construct an effective cell factory that produces a high level of recombinant proteins, a strong promoter is very important. With the study of *Y. lipolytica*, many strong endogenous promoters have been developed for protein expression. The first strong inducible promoter characterized in *Y. lipolytica* was pXPR2, which drives the expression of alkaline extracellular protease (XPR2) [8,9]. However, the full activation of pXPR2 requires high concentrations of peptone and a pH over 6 [10]. When studying the glyoxylate cycle, the β-oxidation of fatty acids, and glycerol metabolism in *Y. lipolytica*, several other inducible promoters were identified, with the acyl-CoA oxidase 2 promoter pPOX2 and 3-oxo-acyl-CoA thiolase promoter pPOT1 showing substantial induction activity [8,11]. Nevertheless, the inducer of pPOX2 and pPOT1 is hydrophobic oleic acid, which affects its practical application [10,11]. Moreover, the constitutive promoter pTEF, which regulates the translation elongation factor-1α gene, has also been used to drive protein expression [12]. Madzak et al. [13] studied the pXPR2 promoter and identified its 105-bp upstream activating sequence (UAS). Subsequent researchers discovered that UAS can act as an enhancer to increase the strength of a promoter [10,14,15]. As a result, a series of strong hybrid promoters harboring multiple UAS elements and a core promoter were developed and have been utilized for heterologous gene expression [8,16]. The powerful hybrid promoter hp4d, created by fusing four tandem UAS of pXPR2 (UAS1B) upstream of the minimum LEU2 promoter (mLeu2), was widely used in *Y. lipolytica* [17]. Despite it, more new promoters remain to be exploited to meet different demands. Previously, studies in our laboratory found that a *Y. lipolytica* recombinant strain secreted a large amount of enolase upon expression of *Rhizomucor miehei* lipase (RML) [18]. Coincidentally, Swietalski et al. [19] also discovered a similar phenomenon when expressing other recombinant proteins. Thus, it can be hypothesized that the enolase promoter (pENO) may have the potential to be developed for the efficient expression of recombinant proteins. However, no research has been conducted to date on the development of pENO for heterologous protein expression in *Y. lipolytica*.

In addition to the promoter, several other factors may also influence the protein expression level in *Y. lipolytica*. Since the codon usage bias of *Y. lipolytica* differs significantly from that of *S. cerevisiae* and *P. pastoris* [20,21], and the codon composition of the target gene may have a great impact on translation efficiency [22], codon optimization was utilized and successfully improved the level of recombinant protein production in *Y. lipolytica* [18,23]. Gene dosage is also a key factor that may affect expression levels, and a common approach used to enhance heterologous protein expression is to increase gene copy number [24,25]. Additionally, protein folding also has a greater effect on recombinant protein expression. The accumulation of unfolded proteins in the endoplasmic reticulum (ER) would cause the unfolded protein response, which would then result in up-regulating some genes involved in protein folding to relieve ER stress, such as the transcriptional activator Hac1, protein disulfide isomerase (Pdi), and chaperone Kar2 [26,27,28,29]. Until now, overexpression of these helper proteins to assist protein folding has been widely used to enhance protein expression in many yeast species, such as *S. cerevisiae* or *P. pastoris*, but has remained relatively rare in *Y. lipolytica* [30]. 

In order to employ genetic engineering strategies to construct high-yield recombinant strains, multiple rounds of gene integration in a single strain are often required. However, the availability of selection markers for *Y. lipolytica* is limited [5], and the use of antibiotic markers may be detrimental to its GRAS status, which makes it difficult to perform multiple genetic modifications in *Y. lipolytica*. To address this problem, a method based on the Cre/*lox*P recombination system that could achieve post-transformational marker rescue was widely used in *Y. lipolytica* [24,31,32]. The approach involves integrating a target fragment with a marker gene flanked by two *lox*P sites into the host genome, followed by introducing a plasmid with a *cre* gene cassette via a second transformation to eliminate the marker gene by Cre/*lox*P recombination. Then, a subsequent culture step is needed to move the *cre*-expressing vector [31]. Obviously, markerless integration using this method requires two rounds of transformation with two different selection markers, which is time-consuming and laborious. Therefore, there is still a need to develop more convenient strategy to achieve markerless integration in *Y. lipolytica*.

Although *Y. lipolytica* has been widely used as an expression platform for the efficient production of numerous recombinant proteins [5,30], to our knowledge, *Thermomyces lanuginosus* lipase (TLL), an important thermostable lipase used in various industrial areas [33], has not been efficiently expressed in *Y. lipolytica*. Based on the above analyses, in this study, 11 truncated fragments upstream of the enolase gene were first employed to identify the pENO promoter and its strength, and the enhanced green fluorescent protein gene (*egfp*) was used as a reporter. Then, seven of the truncated promoters were selected to examine their ability for driving TLL expression. Following this, a set of enolase hybrid promoters was constructed to enhance the expression of TLL. Additionally, a new self-excisable vector for markerless integration was designed. Using this tool, the expression of TLL was further enhanced by optimizing gene dosage and co-expressing the helper proteins Hac1, Pdi, and Kar2.

## 2. Results

### 2.1. Identification of the Core Region of Enolase Promoter

**Figure 1:**
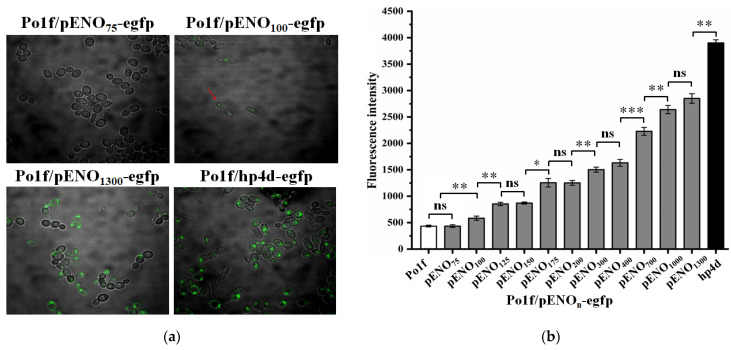
eGFP fluorescence of recombinant strains Po1f/pENO_n_-egfp harboring different truncated enolase promoters pENO_n_ (n = 75, 100, 125, 150, 175, 200, 300, 400, 700, 1000, and 1300 bp). (**a**) Fluorescence detection by laser confocal microscopy. The red arrow represents a cell with fluorescence; (**b**) fluorescence intensity analysis by flow cytometry. Statistical significance was calculated by unpaired T-test analysis. ns: no significant difference; *: *p* < 0.05; **: *p* < 0.01; ***: *p* < 0.001.

To identify the enolase promoter, the plasmids pENO_n_-egfp (n = 75, 100, 125, 150, 175, 200, 300, 400, 700, 1000, and 1300 bp) and hp4d-egfp (control) were introduced into Po1f to construct strains with eGFP under the control of various truncated enolase promoters. Due to the large quantities of extracellular enolase found during fermentation in BMSY medium [18], this medium was also used here to study the enolase promoter. After fermentation, the eGFP fluorescence of Po1f/pENO_n_-egfp (n = 75, 100, 125, 150, 175, 200, 300, 400, 700, 1000, and 1300 bp) was assayed by confocal microscopy and flow cytometry. As shown in Figure 1, no fluorescence was observed as the promoter length of enolase was 75 bp, while the promoter length was above 100 bp, eGFP fluorescence was clearly visible, and the intensity increased significantly with the increase in the promoter length.

**Figure 2:**
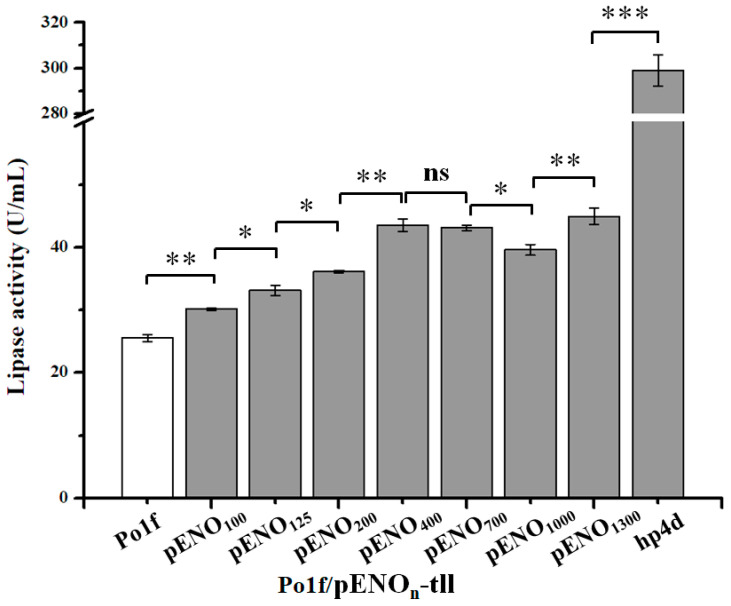
TLL activity of recombinant strains Po1f/pENO_n_-tll harboring different truncated enolase promoter pENO_n_ (n = 100, 125, 200, 400, 700, 1000, and 1300 bp). Statistical significance was calculated by unpaired T-test analysis. ns: no significant difference; *: *p* < 0.05; **: *p* < 0.01; ***: *p* < 0.001.

To determine whether the enolase promoter has the potential to be used for expression of the recombinant protein, seven enolase promoters with varying lengths (100, 125, 200, 400, 700, 1000, and 1300 bp) were further selected for TLL expression. As seen in Figure 2, TLL was successfully expressed using these enolase truncated promoters, and TLL activity increased clearly as the promoter length was increased between 100 and 400 bp. However, unlike the expression of eGFP, further increases in promoter length did not result in a rise in TLL activity. Obviously, the different truncated enolase promoters could be used to drive the secreted expression of exogenous proteins.

### 2.2. Engineering Enolase Hybrid Promoter to Increase Promoter Strength

**Figure 3:**
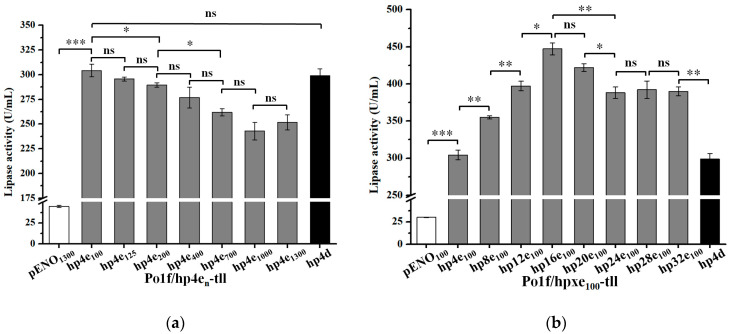
TLL activity of recombinant strains (**a**) Po1f/hp4e_n_-tll (n = 100, 125, 200, 400, 700, 1000, and 1300 bp) and (**b**) Po1f/hpxe_100_-tll (x = 8, 12, 16, 20, 24, 28, and 32). Statistical significance was calculated by unpaired T-test analysis. ns: no significant difference; *: *p* < 0.05; **: *p* < 0.01; ***: *p* < 0.001.

Numerous promoters were strengthened by the upstream fusion of multiple UAS elements [8,14,16,17]. To investigate whether the enolase promoter could also be enhanced using a similar strategy, several enolase hybrid promoters hp4e_n_ (n = 100, 125, 200, 400, 700, 1000, and 1300 bp) were constructed by introducing a four UAS1B sequence upstream of the corresponding truncated promoter and then used for TLL expression. The experimental results revealed that the fusion of four UAS1B upstream of different enolase promoters all had a significant positive effect on TLL expression (Figure 3a). Interestingly, the shorter the truncated enolase promoter, the greater the increase in TLL expression. This may be due to the fact that the distance between the UAS and the core promoter has an effect on the strength of the promoter [34]. As a result, the strain Po1f/hp4e_100_-tll harboring hp4e_100_ hybrid promoter showed the highest TLL activity (305 U/mL), which was comparable to that of the strain Po1f/hp4d-tll containing the commonly used hp4d promoter. These results suggested that UAS1B can also be used to increase the strength of the enolase promoter.

To examine whether an increase in the copy number of UAS1B could further enhance the strength of the enolase promoter, based on hp4e_100_, a series of enolase hybrid promoters hpxe_100_ (x = 8, 12, 16, 20, 24, 28, and 32) harboring varying copies of UAS1B were created via the biobrick method, as previously reported [18,35]. As expected, the strength of enolase hybrid promoter was further improved, the results of TLL expression under the control of these hybrid promoters are shown in Figure 3b. When the copy number of UAS1B did not exceed 16, the TLL activities of the strains were gradually enhanced as the UAS1B dosage was increased. However, an excessive increase in the copy number of UAS1B resulted in no further improvement in TLL activity, but rather a decrease. The recombinant strain Po1f/hp16e_100_-tll harboring the hp16e_100_ enolase hybrid promoter showed the highest lipase activity of 447 U/mL, which was 14.9-fold higher than that of the control strain Po1f/pENO_100_-tll (30 U/mL), suggesting that upstream fusion of multi-copies of UAS1B is also an effective strategy to increase the strength of enolase promoter.

### 2.3. Construction of a Self-Excising Vector for Optimizing the Gene Dosage of Tll

**Figure 4:**
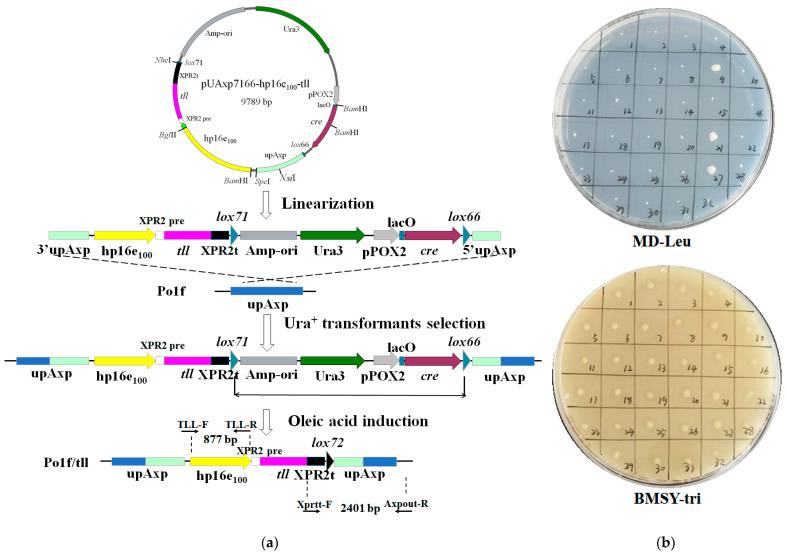
(**a**) Schematic diagram of markerless integration using the self-excisable vector pUAxp7166-hp16e_100_-tll. DNA elements: upAXP, upstream homologous fragment of the acid extracellular protease gene (*axp1*); XPR2 pre, XPR2 signal peptide; XPR2t, *xpr2* gene terminator; Amp-ori, ampicillin resistance gene and origin of replication in *E. coli*; (**b**) A total of 32 recombinants were randomly selected and inoculated onto MD-Leu and BMSY-tri plates to screen positive colonies after the marker-free integration.

With the aim of further enhancing the TLL expression level, the strategy of optimizing gene dosage was employed to achieve this goal. For this purpose, a new self-excising vector, pUAxp7166-hp16e_100_-tll, allowing gene marker-free integration in *Y. lipolytica*, was first designed and constructed. The plasmid harbors a *lox71*-*cre*-*Ura3*-Amp-ori-*lox66* fragment, with the left-arm mutant *lox71* and the right-arm mutant *lox66* sites oriented in the same direction. The *cre* gene is regulated by the inducible promoter pPOX2, and the lac operator gene of the lactose operon (lacO) is introduced upstream of the *cre* gene to block the leakage expression of Cre in *Escherichia coli* [36]. The processes for markerless integration of the *tll* gene using this plasmid are shown in Figure 4a.

To test whether the self-excising vector could achieve marker-free integration, pUAxp7166-hp16e_100_-tll was linearized within the upAxp homologous fragment and then transformed into Po1f. The transformants were first incubated twice in MD-Leu liquid medium without uracil to screen Ura^+^ transformants. Following that, the Ura^+^ recombinants were then inoculated into MO-Leu-Ura medium, which uses oleic acid as the sole carbon source. In this circumstance, pPOX2 was substantially induced and then activated Cre expression. After 24 h of induction in MO-Leu-Ura medium, the recombinants were streaked onto an YPD plate to isolate single colonies. Then, 32 clones were randomly picked to both MD-Leu and BMSY-tri plates to examine the *Ura3* phenotype. As seen in Figure 4b, all clones grew well in the BMSY-tri plate, while 29 of them could not grow on the MD-Leu plate, hinting that the *ura3* marker gene was efficiently removed by Cre recombination. To further prove this, 19 Ura3^-^ recombinants were randomly selected for identification by genomic PCR using primer pair XprTT-F2/Axpout-R. As a result, all recombinants were able to obtain the target fragment of about 2400 bp (Figure S1), and the PCR products were then confirmed by DNA sequencing. As expected, the *ura3* marker gene was successfully removed by the desired recombination event, and a double-mutant *lox72* site was retained in the genome. It is difficult for the *lox72* site to participate in subsequent recombination reactions [36,37], which greatly reduces the risk of gene rearrangement when using this self-excising vector for repeat gene integration in the same host. Furthermore, ten positive strains were further selected for verification by PCR with the primers TLL-F and TLL-R, and all of these strains contain the target *tll* gene (Figure S1). These results showed that the vector pUAxp7166-hp16e_100_-tll achieved efficient marker-free integration of the *tll* gene in *Y. lipolytica*, and the resulting strain was named Po1f/tll.

To obtain strains containing multiple copies of the *tll* gene, the vector pUAxp7166-hp16e_100_-tll was re-transformed into the strain Po1f/tll again. After transformation and marker rescue, the strain Po1f/2tll, harboring two copies of *tll* gene, was constructed. Similarly, the strains Po1f/3tll and Po1f/4tll were created in turn. The gene copy number of these strains were verified by RT-PCR, and the results showed that the strains harbored the desired number of copies of the *tll* gene (Table 1). 

**Figure 5:**
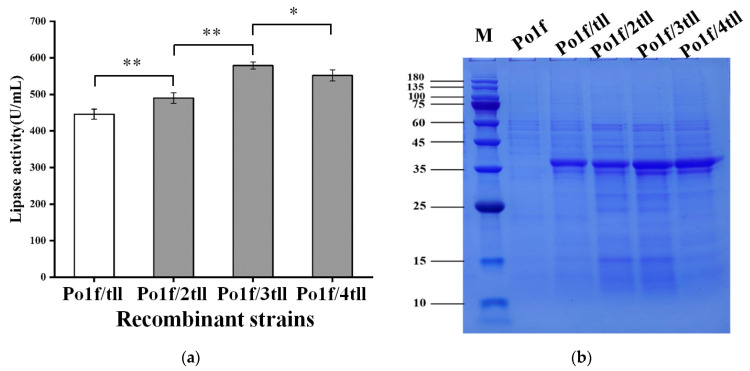
Cultivation results of the recombinant strains Po1f/ntll (n = 1, 2, 3, and 4) harboring different copies of *tll* gene in shaking flasks. (**a**) TLL activity; Statistical significance was calculated by unpaired T-test analysis. *: *p* < 0.05; **: *p* < 0.01. (**b**) SDS-PAGE analysis.

Subsequently, recombinant strains Po1f/ntll (n = 1, 2, 3, and 4) were fermented in shaking flasks, and the fermentation supernatants were used for lipase assays and sodium dodecyl sulfate polyacrylamide gel electrophoresis (SDS-PAGE) analyses. As displayed in Figure 5a and Table S1, the TLL expression level was improved as the *tll* gene copy number increased from one to three. However, when the *tll* copy number was further enhanced to four, the expression of TLL did not increase and even decreased to some extent. SDS-PAGE results (Figure 5b) showed that two protein bands with molecular masses slightly larger than 35 kDa were clearly separated, and the concentration of the protein bands in the different strains was consistent with their TLL activity. The fermented supernatant was further treated with Endo H (NEB, America). After deglycosylation, the larger band disappeared and the smaller band became more intense (Figure S2a), suggesting that the larger band was caused by glycosylation modifications. Subsequently, the protein band was validated by mass spectrometry. The results showed that the peptides matched the TLL protein sequence (Figure S2b), confirming that the two bands were all target protein TLL. Thus, the strain Po1f/3tll with three copies of the *tll* gene exhibited the highest TLL activity and maximum extracellular protein concentration of 579 U/mL and 0.438 g/L, which were approximately 30% and 43% higher than those of the single-copy strain Po1f/tll, respectively.

### 2.4. Shaking Flask Culture Optimization for Po1f/3tll

Several fermentation parameters were optimized in shaking flasks to favor the efficient expression of TLL (Figure S3). Obviously, 4% (*m*/*v*) of D-sorbitol was more suitable for the TLL expression. Based on this, when 2% (*v*/*v*) strain Po1f/3tll was inoculated into 500 mL shake flasks containing 30 mL BMSY medium and fermented at pH 6.0 for 120 h, the maximum lipase activity and the total protein concentration of the strain were increased to 720 U/mL and 0.530 g/L, respectively.

### 2.5. Co-Expressing Helper Genes to Enhance TLL Expression

**Figure 6:**
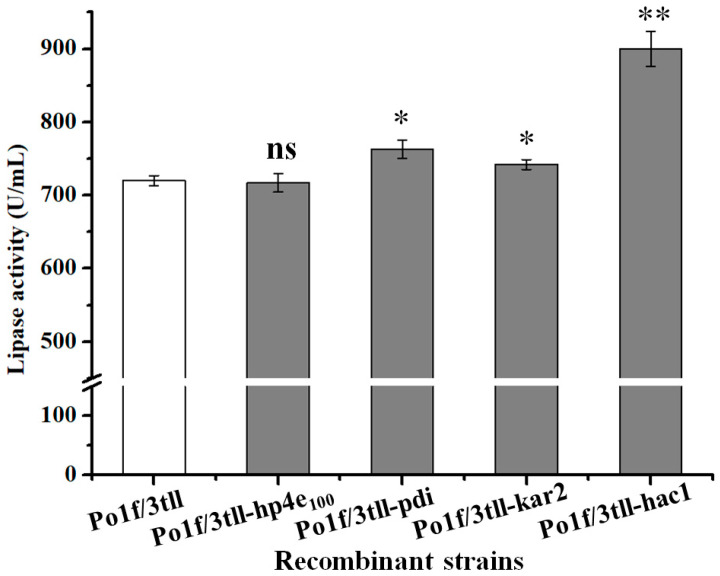
Effect of co-expressing a single folding-related helper protein on TLL production in the strain Po1f/3tll. Statistical significance was calculated by unpaired T-test analysis. ns: no significant difference; *: *p* < 0.05; **: *p* < 0.01.

In an attempt to further increase the expression level of TLL, three helper proteins (Hac1, Pdi, and Kar2) that aid in protein folding were employed to co-express with TLL. For this purpose, the vectors pUXpr7166-hp4e_100_ (control), pUXpr7166-hac1, pUXpr7166-kar2, and pUXpr7166-pdi harboring different helper genes under the control of hp4e_100_ enolase hybrid promoter were linearized within the upXpr homologous fragments and then introduced into the strain Po1f/3tll, respectively. After marker rescue, recombinant strains were cultured in 500 mL shaking flasks. As shown in Figure 6, overexpression of Kar2 and Pdi resulted in a slight increase in the production of TLL, with the TLL activities reaching 742 U/mL and 763 U/mL in the strains Po1f/3tll-kar2 and Po1f/3tll-pdi, respectively. Co-expression of Hac1 led to a 25% improvement in TLL activity, and the recombinant strain Po1f/3tll-hac1 exhibited lipase activity and an extracellular protein concentration of 900 U/mL and 0.620 g/L, respectively. The results indicating that the folding-related proteins had a positive effect on TLL expression.

**Figure 7:**
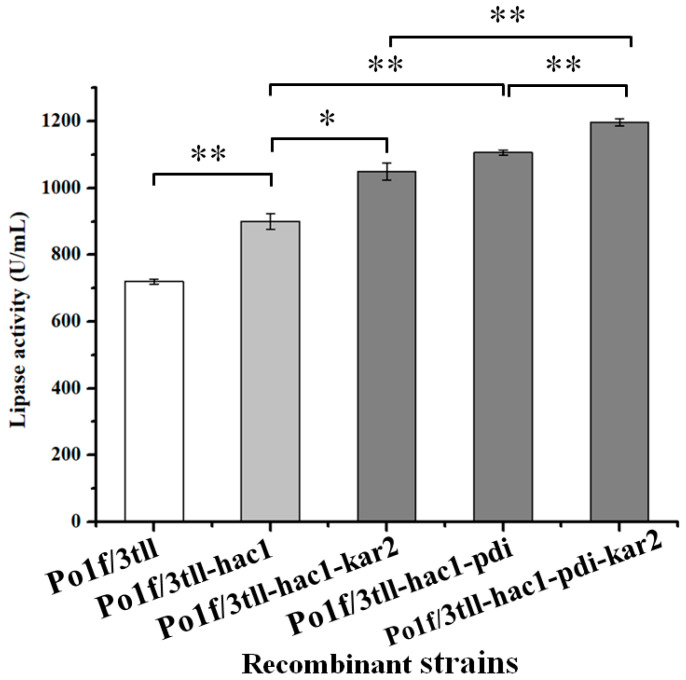
Effect of co-expressing multiple folding-related helper proteins on TLL production in the strain Po1f/3tll. Statistical significance was calculated by unpaired T-test analysis. *: *p* < 0.05; **: *p* < 0.01.

To investigate the influence of co-expression of multiple helper proteins on TLL expression, the plasmids pUXpr7166-pdi and pUXpr7166-kar2 were used to transform the best-performing engineered Po1f/3tll-hac1 to produce recombinant strains Po1f/3tll-hac1-pdi and Po1f/3tll-hac1-kar2, respectively. The lipase activity of the different strains was measured after 120 h of fermentation (Figure 7), the activities of the strains Po1f/3tll-hac1-pdi and Po1f/3tll-hac1-kar2, respectively, were enhanced to 1107 U/mL and 1050 U/mL. Furthermore, the plasmid pUXpr7166-kar2 was further introduced into Po1f/3tll-hac1-pdi so that these three helper proteins were co-expressed with TLL. As a result, a maximum TLL activity of 1197 U/mL was gained in the strain Po1f/3tll-hac1-pdi-kar2, which was 66% higher than that of the strain Po1f/3tll. Additionally, the extracellular protein concentration of Po1f/3tll-hac1-pdi-kar2 further increased to 0.779 g/L. These results suggest that Hac1, Pdi, and Kar2 had a synergistic effect on TLL expression.

## 3. Discussion

Previous research discovered that *Y. lipolytica* secreted large amounts of enolase when it expressed recombinant proteins [18,19], hinting that the promoter of enolase might be strong. However, the enolase promoter has not been developed for the secretory expression of recombinant proteins. With the aim of developing the enolase promoter for efficient expression of recombinant proteins, the enolase promoter was first identified using a common strategy [16,38] by the expression of an *egfp* reporter gene regulated by 11 truncated promoters with different lengths (75–1300 bp). The eGFP expression experiments revealed that the 100-bp pENO_100_ was capable of driving protein expression, with longer promoters exhibiting increased activity, indicating that the 100 bp upstream fragment of the enolase gene contains the core promoter region required for promoter activity, and the region between 100 bp and 1300 bp may contain multiple elements that can enhance promoter activity. Following that, several truncated enolase promoters (100–1300 bp) were selected for attempts to express TLL. The results revealed that TLL was successfully expressed. Similar to eGFP, the expression of TLL gradually improved as the length of the enolase promoter increased within 400 bp. However, contrary to the expression of eGFP, further increasing the length of the promoter (700–1300 bp) did not enhance the expression of TLL. This is similar to previously reported findings that stronger promoters do not always lead to higher protein yields when expressing proteins using different modalities (intracellularly or extracellularly) [39]. In fact, TLL was secreted extracellularly, whereas eGFP was expressed intracellularly. In addition to the effect of promoter strength, TLL expression may also be influenced by the secretory pathway. Nevertheless, the results demonstrate that the enolase promoter can be used for the expression of recombinant proteins.

Several strong hybrid promoters have been developed by fusing multiple repeats of UAS fragments upstream of the promoter [10,15,17,39]. A similar strategy was adopted to construct several enolase hybrid promoters by fusing UAS1B. As expected, the strength of enolase promoter could also be enhanced by the UAS1B sequence. It is worth mentioning that the effect of UAS1B on the strength of the enolase hybrid promoter depends not only on its distance with the core promoter region but also on its copy number, which is consistent with previous studies [17,34,39]. Among the several engineered enolase hybrid promoters, TLL expression using the hp4e_100_ promoter was comparable to that of hp4d, while strain Po1f/hp16e_100_-tll harboring the optimal hybrid promoter hp16e_100_ achieved an even higher TLL activity of 447 U/mL, which was 14.9-fold higher than that of pENO_100_. By truncating the promoter and constructing hybrid promoters, a series of enolase promoters with different strengths were obtained (Figure 1, Figure 2 and Figure 3). Of these, the truncated promoters pENO_n_ yielded relatively low lipase activity, while promoters hp4e_1300_, hp4e_100_, hp8e_100_, hp12e_100_, and hp16e_100_ resulted in TLL activities ranging from 251 to 447 U/mL at intervals of approximately 50 U/mL, which allowed promoters with different gradients of strength to be applied to fine-tune the protein expression levels when required.

It is well known that gene dosage is one of the key parameters for optimizing the expression of recombinant proteins. To facilitate the optimization of gene dosage to enhance the TLL expression and avoid the introduction of antibiotic gene that affect the GRAS status, a new self-excisable vector based on the Cre/*lox*P recombination system was designed and successfully achieved efficient markerless integration in *Y. lipolytica*. Previously, a Cre/*lox*P-based method has been widely used for marker-free integration in *Y. lipolytica* [31,40]. However, in this traditional approach, an additional vector containing the *cre* gene expression cassette was required to achieve marker rescue, and, therefore, two rounds of transformations were necessary to complete marker-free integration [31]. In contrast, the two *lox* sites (*lox71* and *lox66*) and the *cre* gene expression cassette were combined in the self-excisable vector, and the *cre* gene was under the control of the pPOX2 inducible promoter, allowing marker-free integration with only one transformation. Therefore, the use of the new tool significantly reduces the experimental period and workload compared to traditional methods.

Using this self-excisable vector, recombinant strains carrying one to four copies of *tll* gene were constructed. As a result, three-copy *tll* gene resulted in the highest yield. While the *tll* copy number increased from three to four, the expression level of TLL decreased instead. The reason might be that when *tll* gene dosage was moderately increased, the transcription of *tll* gene increases, resulting in an increase in its expression, while the high gene dosage might have resulted in the excessive aggregation of abnormal proteins in the ER and caused cellular stress, which, in turn, decreased the expression level [2,27,28]. By optimizing the gene dosage, the TLL activity was increased by about 30%, and the recombinant strain Po1f/3tll had a maximum lipase activity of 579 U/mL. Fermentation also has a greater impact on TLL expression of a recombinant strain. After optimization of the fermentation parameters, the TLL activity of the optimal strain Po1f/3tll was further increased to 720 U/mL.

With the aim of further increasing the yield of TLL, the helper proteins Hac1, Pdi, and Kar2, which aid in protein folding [26,29], were selected to co-express with TLL in the strain Po1f/3tll. The fermentation results found that the three folding-related proteins all had a positive effect on TLL expression. Among them, overexpression of Hac1, resulting in 25% improvement in TLL activity, and strain Po1f/3tll-hac1 had a lipase activity of 900 U/mL. To explore whether co-expression of multiple helper proteins was beneficial for the expression of TLL, the helper proteins Kar2 and/or Pdi were transformed into strain Po1f/3tll-hac1 to achieve this goal. Excitingly, the TLL activities were further improved to 1050 U/mL, 1107 U/mL in the strains Po1f/3tll-hac1-pdi, Po1f/3tll-hac1-kar2, respectively. Furthermore, co-expression of Hac1, Pdi, and Kar2 with TLL in the strain Po1f/3tll-hac1-pdi-kar2 reached the highest TLL activity of 1197 U/mL, which was about 66% higher than that of the strain Po1f/3tll. These results showed that the folding-related proteins had a synergistic positive influence on TLL expression. This might be due to the fact that protein folding efficiency was a bottleneck for high-level TLL expression in the Po1f/3tll, while the protein folding efficiency was improved by co-expression of the folding-related proteins, which allowed for a further increase in the expression of TLL. Obviously, the results presented here show that co-expression of folding-related proteins is also a feasible option for improving the heterologous protein production in *Y. lipolytica*.

In conclusion, a series of enolase hybrid promoters with different strengths and a new self-excisable vector for efficient marker-free integration were developed in this study, which extends the toolbox of *Y. lipolytica*. Furthermore, the TLL was successfully expressed in *Y. lipolytica* for the first time and significantly enhanced (39.9-fold) by using an enolase strong hybrid promoter, optimizing gene dosage, and co-expressing multiple folding-related proteins. This combined strategy might also be useful in optimizing the expression of other recombinant proteins in *Y. lipolytica*.

## 4. Materials and Methods

### 4.1. Strains, Plasmids, and Media

*E. coli* Top10 and Top10F’ were commercially obtained from Shanghai Weidi Biotechnology Co., Ltd. (Shanghai, China). Y. lipolytica Po1f (Ura^−^ Leu^−^) [41] was used as hosts. The plasmids hp4d-rml [18], Cre-Y3 [42], Cre-axp1 [42], T-egfp (unpublished), and Mlu-vgb (unpublished) were stored in our laboratory. The T4 DNA ligase (TaKaRa, Dalian, China), PrimeStar HS DNA polymerase (TaKaRa), and ClonExpress II One Step Cloning Kit (Vazyme, Nanjing, China) were used for gene amplification and genome identification. All strains, plasmids, and primers used in this study are, respectively, listed in Tables S2 and S3.

The *E. coli* strains were cultured in Luria-Bertani medium supplemented with 100 μg/mL ampicillin. *Y. lipolytica* strains were grown in yeast extract-peptone-dextrose (YPD), minimal dextrose (MD), minimal oleic acid (MO), buffered sorbitol-complex medium (BMSY), BMSY containing 10 mL/L glyceryl tributyrate (BMSY-tri), according to experimental needs. The media and the cultural conditions have been previously described [18,42]. To meet auxotrophic requirements, leucine (262 mg/L) and/or uracil (22.4 mg/L) were added as needed.

### 4.2. Vector Construction

The *egfp* gene was amplified from T-egfp using primers egfp-F and egfp-R then subcloned to *Hind*III/*Kpn*I-digested hp4d-rml to produce hp4d-egfp (Figure S4A). The various lengths of enolase (YALI0F16819g) promoter fragments were cloned from the genomic DNA of Po1f using primer pENOn-F1 and pENO-R1, the PCR products were, respectively, digested with *Bam*HI and *Hind*III and inserted into the *Bam*HI/*Hind*III-opened plasmid hp4d-egfp, generating pENO_n_-egfp (n = 75, 100, 125, 150, 175, 200, 300, 400, 700, 1000, and 1300 bp) (Figure S4B) encoding eGFP under the control of desired enolase promoters. The mature *tll* gene (Genbank accession no. AF054513.1) lacking the signal sequence was synthesized according to the codon usage database of *Y. lipolytica* [20], which was then amplified by the primer pair TLL-F/TLL-R. The PCR products were inserted into *Sfi*I/*Kpn*I-digested hp4d-rml by homologous recombination (HR) to form hp4d-tll (Figure S4C). Subsequently, the XPR2 pre and *tll* gene was isolated from hp4d-tll by double digestion with *Hind*III and *Kpn*I, the digested product then, respectively, cloned into plasmids pENO_n_-egfp to replace the *egfp* gene, generating pENO_n_-tll (n = 100, 125, 200, 400, 700, 1000, and 1300 bp) (Figure S4D).

To construct the enolase hybrid promoter, the desired promoters were, respectively, amplified from the genomic DNA of Po1f using pENOn-F2 (n = 100, 125, 200, 400, 700, 1000, and 1300 bp) and pENO-R1, followed by digestion with *Bgl*II and *Hind*III to obtain pENOn. In parallel, the plasmid hp4d-tll was double digested with *Sal*I/*Bgl*II to isolate the four-UAS1B sequence. Then, pENOn and four-UAS1B were ligated into *Sal*I/*Hind*III-digested hp4d-tll, replacing the mLeu2 promoter to form hp4e_n_-tll (n = 100, 125, 200, 400, 700, 1000, and 1300 bp), respectively (Figure S4E). Based on hp4e_100_-tll, hpxe_100_-tll (x = 8, 12, 16, 20, 24, 28, and 32) containing different copies of UAS1B were constructed via the biobrick method as previously reported (Figure S5) [18].

Using plasmid Cre-Y3 as a template, the fragments lip2TT-*lox66* and Xpr2TT-*lox71* were amplified using primer pairs dwCre-F/lipTT66-R1 and XprTT-F/Xprlox71-R, respectively. The segment upAxp was cloned from the genomic DNA of Po1f using primers 66upAxp-F and upAxp-R, the hp4d promoter was amplified from hp4d-rml by hp4d-F/Xpr2pre-R, and the *Rhizopus oryzae* lipase (ROL) gene was obtained from Cre-axp1 by PCR using primers ROL-F and kpnROL-R. The above five products were ligated by overlap PCR to produce lip2TT-*lox66*-upAxp-hp4d-rol-XprTT-*lox71*, and the product was inserted into *Avr*II/*Eco*RI-digested Cre-Y3 by HR to generate pUAxp7166-rol. Then, the *tll* gene cassette was isolated from hp12e_100_-tll by double digestion with *Bam*HI and *Nhe*I, followed by subcloned into the *Bgl*II/*Nhe*I-digested pUAxp7166-rol to obtain pUAxp7166-hp16e_100_-tll (Figure S6A).

The *Vitreoscilla* hemoglobin gene (*vgb*) from *Vitreoscilla stercoraria* was cloned using Yvgb-F and Yvgb-R, and Mlu-vgb as the template; using pUAxp7166-rol as a template, the fragments mLeu and Xpr2TT were amplified using primer pairs mLeu-F/mLeu-R and XprTT-F2/Ampup-R, respectively. The three PCR products were ligated by overlap PCR then subcloned into *Bgl*II-*Nhe*I sites of pUAxp7166-rol, forming pUAxp7166-vgb. The fragments lip2t-*lox66* was amplified by primer pairs dwCre-F/lipTT66-R2 using pUAxp7166-rol as the template, and the fragment upXpr was cloned from the chromosomal DNA of Po1f using primers 66upXpr-F and upXpr-R. These two products were ligated by overlap PCR then inserted into pUAxp7166-vgb using *Avr*II-*Spe*I sites to replace the homologous fragment upAxp, generating pUXpr7166-vgb. Using Po1f genomic DNA as the template, the fragments pENO_100_-*Bgl*II, pENO_100_-*Bam*HI, pENO_100_-*Not*I, *kar2*, and *pdi* were, respectively, cloned via primer pairs pENO_100_-F3/pENO-R2, pENO_100_-F4/pENO-R2, pENO_100_-F3/pENO-R3, Kar2-F/Kar2-R and Pdi-F/Pdi-R. Additionally, the *hac1* gene without intron was amplified from Cre-Y3-hac1 using primer pair Hac1-F and Hac1-R. Subsequently, the fragments pENO_100_-*Bgl*II and *hac1* gene were ligated by overlap PCR to produce pENO_100_-hac1, then pENO_100_-*Not*I and pENO_100_-hac1 were inserted into pUXpr7166-vgb via *Bgl*II-*Not*I sites, obtaining pUXpr7166-hp4e_100_ and pUXpr7166-hac1, respectively. Similarly, the genes *kar2* and *pdi* were ligated with pENO_100_-*Bam*HI then subcloned into *Bam*HI-*Not*I sites of pUXpr7166-vgb, forming pUXpr7166-kar2 and pUXpr7166-pdi, respectively. The schematic diagram of the pUXpr7166-type plasmids is presented in Figure S6B.

Gene synthetic and DNA sequencing were performed by Tsingke Biological Technology Co. (Wuhan, China).

### 4.3. Transformation of Y. lipolytica

The plasmids pUAxp7166-hp16e_100_-tll, pUXpr7166-hp4e_100_, pUXpr7166-hac1, pUXpr7166-kar2, and pUXpr7166-type were, respectively, linearized by *Nsi*I and transformed into Po1f competent cells. The Ura^+^ transformants were enriched twice in 5 mL of MD-Leu liquid medium, then transferred into MO-Leu-Ura medium to induce Cre expression, followed by the cells being streaked onto YPD plates. The phenotype of isolated colonies was identified using MD-Leu and BMSY-tri plates. Other plasmids were linearized with *Apa*I then transformed into Po1f, transformants were screened in MD-Leu plate. The plasmid transformation was performed according to the protocol described by Chen et al. [43].

### 4.4. Shaking Flask Fermentation and Optimization

After transformation, the transformants were further verified by genome PCR. Subsequently, the recombinant strains were cultured in 5 mL YPD medium overnight and then inoculated into 50 mL of BMSY medium at 28 °C for 120 h. In order to optimize the fermentation conditions in a shaking flask, different incubation parameters were investigated, including D-sorbitol concentration, inoculation density, initial pH, culture medium volume, and fermentation time.

### 4.5. Fluorescence Analysis

The cells were washed 3 times with phosphate-buffered saline (PBS) and then re-suspended in PBS solution. Fluorescence images were obtained with an Olympus FV3000 laser scanning confocal microscope (Olympus, Tokyo, Japan), and eGFP was excited by a 488 nm confocal laser. Additionally, fluorescence acquisition was conducted using a flow cytometer (Cytoflex, Beckman Coulter, Brea, CA, USA) in the FITC channel, with 1 × 10^5^ cells per sample were acquired for each collection.

### 4.6. Enzyme Assay and Total Protein Concentration

The TLL activity was determined by titration using olive oil as a substrate [44]. A spectrophotometric approach was used to quantify total protein concentration using bovine serum albumen as a standard [45]. Measurements were repeated three times for every sample.

### 4.7. Determination of Gene Copy Number

The real-time PCR (RT-PCR) was performed using a StepOnePlus instrument with StepOne software version 2.3 (Applied Biosystems, Foster City, CA, USA) as described previously [35]. The standard plasmids hp4d-tll and pMD19-act1 [18] were used to establish the standard curve of target or reference genes, respectively. The gene copy number was calculated via absolute quantification by the method described by Abad et al. [46].

### 4.8. SDS-PAGE Analysis and Mass Spectrometry

Thirty microliters of fermentation supernatant from different strains were analyzed by SDS-PAGE. SDS-PAGE was performed with a 12% separating polyacrylamide gel in a vertical mini gel apparatus (Bio-Rad, Hercules, CA, USA). The proteins in SDS-PAGE gel were stained with Coomassie Brilliant Blue R-250 (Amresco, Solon, OH, USA). The target protein was further identified by mass spectrometry at ProtTech Inc (Suzhou, China).

### 4.9. Statistical Analyses

The results of the study were shown as mean ± standard. Statistical significance was calculated by unpaired T-test analysis. *p* < 0.05 was considered statistically significant. ns: no significant difference; *: *p* < 0.05; **: *p* < 0.01; ***: *p* < 0.001.

## Figures and Tables

**Table 1:** Recombinant strains with different copy numbers of the *tll* gene.

Strains	Gene Copy Number
Po1f (control)	0
Po1f/tll	1
Po1f/2tll	2
Po1f/3tll	3
Po1f/4tll	4

## Data Availability

Data are contained within the article and the Supplementary Material.

## Supplementary Materials

The supporting information can be downloaded at: https://www.mdpi.com/article/10.3390/ijms24010719/s1.
