# Supplementary material for: Employing Engineered Enolase Promoter for Efficient Expression of Thermomyces lanuginosus Lipase in Yarrowia lipolytica via a Self-Excisable Vector
Source: Int J Mol Sci. 2022 Dec 31;24(1):719. doi: 10.3390/ijms24010719 (PMC9821249; doi:10.3390/ijms24010719)
Supplement: Supplementary file 1 [file ijms-24-00719-s001.zip › Supplementary Materials.pdf]

Supplementary Materials

Employing engineered enolase promoter for efficient expression of *Thermomyces lanuginosus* lipase in *Yarrowia lipolytica* via a self-excisable vector

Liangcheng Jiao†, Wenjuan Li†, Yunchong Li, Qinghua Zhou, Mengqin Zhu, Guowei Zhao, Houjin Zhang, Yunjun Yan*

Key Laboratory of Molecular Biophysics of the Ministry of Education, College of Life Science and Technology, Huazhong University of Science and Technology, Wuhan 430074, P. R. China

* Correspondence: yanyunjun@hust.edu.cn; Tel/Fax: +86-27-87792213;

†These authors contributed equally to this work.

Supplementary Tables

Table S1. the extracellular protein concentration of recombinant strains.

Strains	the extracellular protein concentration (g/L)	Annotation
Po1f	0.129 ± 0.003	-
Po1f/tll	0.307 ± 0.018	-
Po1f/2tll	0.380 ± 0.012	-
Po1f/3tll	0.438 ± 0.025	Before fermentation conditions were optimized
Po1f/4tll	0.422 ± 0.010	-
Po1f/3tll	0.530 ± 0.024	After optimization of fermentation conditions
Po1f/3tll-hp4e$_{100}$	0.523 ± 0.016	-
Po1f/3tll-hac1	0.620 ± 0.038	-
Po1f/3tll-kar2	0.560 ± 0.020	-
Po1f/3tll-pdi	0.572 ± 0.024	-
Po1f/3tll-hac1-kar2	0.720 ± 0.027	-
Po1f/3tll-hac1-pdi	0.724 ± 0.016	-
Po1f/3tll-hac1-pdi-kar2	0.779 ± 0.024	-

Table S2. Strains and plasmids used in this study.

Plasmids/strains	Description	Reference or source
**Plasmids**		
hp4d-rml	pINA 1296 derivative, harboring *rml* gene under the control of hp4d promoter	[18]
Cre-Y3	harboring *cre* expression cassette under the control of pPOX2 promoter	[42]
Cre-axp1	harboring *rol* gene expression cassette	[42]
T-egfp	harboring *egfp* gene expression cassette	Our laboratory
Mlu-vgb	harboring *vgb* gene expression cassette	Our laboratory
pMD19-act1	harboring *act1* gene expression cassette	Our laboratory
hp4d-egfp	hp4d-rml derivative, harboring *egfp* gene under the control of hp4d promoter	This study
hp4d-tll	hp4d-rml derivative, harboring *tll* gene under the control of hp4d promoter	This study
pENOn-egfp (n = 75, 100, 125, 150, 175, 200, 300, 400, 700, 1000, and 1300 bp)	hp4d-egfp derivative, harboring *egfp* gene under the control of different length of enolase promoter	This study
pENOn-tll (n = 100, 125, 200, 400, 700, 1000, and 1300 bp)	pENOn-egfp derivative, harboring *tll* gene under the control of different length of enolase promoter	This study
hp4e_n-tll (n = 100, 125, 200, 400, 700, 1000, and 1300 bp)	hp4d-tll derivative, harboring *tll* gene under the control of different length of enolase hybrid promoter	This study
hpxe_{100}-tll (x = 8, 12, 16, 20, 24, 28, and 32)	hp4e_{100}-tll derivative, harboring *tll* gene under the control of enolase hybrid promoter containing different copies of UAS1B	This study
pUAxp7166-hp16e_{100}-tll	Cre-Y3 and hp16e_{100}-tll derivative, harboring upAxp homologous fragment, a self-excising vector for *tll* gene markerless integration	This study
pUXpr7166-hp4e_{100}	pUAxp7166-hp16e_{100}-tll derivative, harboring upXpr homologous fragment	This study
pUXpr7166-hac1	pUXpr7166-hp4e_{100} derivative, a self-excising vector for *hac1* gene markerless integration	This study
pUXpr7166-kar2	pUXpr7166-hp4e_{100} derivative, a self-excising vector for *kar2* gene markerless integration	This study
pUXpr7166-pdi	pUXpr7166-hp4e_{100} derivative, a self-excising vector for *pdi* gene markerless integration	This study

Strains

Po1f	*MatA*, *leu2-270*, *ura3-302*, *xpr2-322*, *axp1-2*, Leu⁻ , Ura⁻ , ΔAEP, ΔAXP	[41]
Po1f/pENO$_n$-egfp (n = 75, 100, 125, 150, 175, 200, 300, 400, 700, 1000, and 1300 bp)	eGFP expressed in Po1f using pENO$_n$-egfp	This study
Po1f/hp4d-egfp	eGFP expressed in Po1f using hp4d-egfp	This study
Po1f/pENO$_n$-tll (n = 100, 125, 200, 400, 700, 1000, and 1300 bp)	TLL expressed in Po1f using pENO$_n$-tll	This study
Po1f/hp4d-tll	TLL expressed in Po1f using hp4d-tll	This study
Po1f/hp4e$_n$-tll (n = 100, 125, 200, 400, 700, 1000, and 1300 bp)	TLL expressed in Po1f using hp4e$_n$-tll	This study
Po1f/hpxe$_{100}$-tll (x = 8, 12, 16, 20, 24, 28, and 32)	TLL expressed in Po1f using hpxe$_{100}$-tll	This study
Po1f/1tll	Po1f derivative, harboring 1 copy of *tll* expression cassette	This study
Po1f/2tll	Po1f/1tll derivative, harboring 2 copies of *tll* expression cassettes	This study
Po1f/3tll	Po1f/2tll derivative, harboring 3 copies of *tll* expression cassettes	This study
Po1f/4tll	Po1f/3tll derivative, harboring 4 copies of *tll* expression cassettes	This study
Po1f/3tll-hp4e$_{100}$	Po1f/3tll derivative, harboring an empty expression cassette	This study
Po1f/3tll-hac1	Po1f/3tll harboring *hac1* gene cassette	This study
Po1f/3tll-kar2	Po1f/3tll harboring *kar2* gene cassette	This study
Po1f/3tll-pdi	Po1f/3tll harboring *pdi* gene cassette	This study
Po1f/3tll-hac1-pdi	Po1f/3tll harboring *hac1* gene cassette and *pdi* gene cassette	This study
Po1f/3tll-hac1-kar2	Po1f/3tll harboring *hac1* gene cassette and *kar2* gene cassette	This study
Po1f/3tll-hac1-pdi-kar2	Po1f/3tll harboring *hac1* gene cassette, *pdi* gene cassette, and *kar2* gene cassette	This study

Table S3. Primers used in this study.

Primers	Sequence (5'-3')	Annotation
egfp-F	CCCAAGCTTATGGTGAGCAAGGGCGAGGAG	*Hind*III site (underlined)
egfp-R	GGGGTACCTTACTTGTACAGCTCGTCC	*Kpn*I site (underlined)
TLL-F	ACCGCCTTTACTATTCTCACGGCCGTTCTGGCCATGGAGGTCTCCCAGGACCTGTTC	*Sfi*I site (underlined)
TLL-R	CGGCAACGTGGGGACAGGCCATGGAGGTACCTTACAGGCAGGTGCCGATCAGGCCG	*Kpn*I site (underlined)
pENO$_{75}$-F1	GAGCACCGCCGCCGCAAGGAATGGTGGATCCTAAAAGGAGCAATCCCCCACTAG	*Bam*HI site (underlined)
pENO$_{100}$-F1	GAGCACCGCCGCCGCAAGGAATGGTGGATCCAGCGCAATTGGGTTTGCATCATGTA	*Bam*HI site (underlined)
pENO$_{125}$-F1	GAGCACCGCCGCCGCAAGGAATGGTGGATCCGATGACTATTTGGGACCAATCCAA	*Bam*HI site (underlined)
pENO$_{150}$-F1	GAGCACCGCCGCCGCAAGGAATGGTGGATCCTGACGAAAGCACCCCCCTTTGTACA	*Bam*HI site (underlined)
pENO$_{175}$-F1	GAGCACCGCCGCCGCAAGGAATGGTGGATCCCCCCCAATGGCTTTTAACTTTCGAA	*Bam*HI site (underlined)
pENO$_{200}$-F1	GAGCACCGCCGCCGCAAGGAATGGTGGATCCAGTCCTTCAACAATCCATCTCACC	*Bam*HI site (underlined)
pENO$_{300}$-F1	GAGCACCGCCGCCGCAAGGAATGGTGGATCCGTATTTGAGAGCAAGTTTGGGATT	*Bam*HI site (underlined)
pENO$_{400}$-F1	GAGCACCGCCGCCGCAAGGAATGGTGGATCCTTTCACTTGCCAGAACTCTAAGCG	*Bam*HI site (underlined)
pENO$_{700}$-F1	GAGCACCGCCGCCGCAAGGAATGGTGGATCCGTCCAGTGCAACCCAGACAGCTGAG	*Bam*HI site (underlined)
pENO$_{1000}$-F1	GAGCACCGCCGCCGCAAGGAATGGTGGATCCCCATGGTGCGTGGAGGCTTTGGCA	*Bam*HI site (underlined)
pENO$_{1300}$-F1	GAGCACCGCCGCCGCAAGGAATGGTGGATCCATCTACCTCGGCCCTTGTTTCTTGA	*Bam*HI site (underlined)

pENO-R1	TCCTCGCCCTTGCTCACCATAAGCTTGGTGATAAATGTGTGGTTAGACGGG	*Hind*III site (underlined)
pENO$_{100}$-F2	GGAAGATCTAGCGCAATTGGGTTTGCATCATGTA	*Bgl*II site (underlined)
pENO$_{125}$-F2	GGAAGATCTGATGACTATTTGGGACCAATCCAA	*Bgl*II site (underlined)
pENO$_{200}$-F2	GGAAGATCTAGTCCTTCAACAATCCATCTCACC	*Bgl*II site (underlined)
pENO$_{400}$-F2	GGAAGATCTTTTCACTTGCCAGAACTCTAAGCG	*Bgl*II site (underlined)
pENO$_{700}$-F2	GGAAGATCTGTCCAGTGCAACCCAGACAGCTGAG	*Bgl*II site (underlined)
pENO$_{1000}$-F2	GGAAGATCTCCATGGTGCGTGGAGGCTTTGGCA	*Bgl*II site (underlined)
pENO$_{1300}$-F2	GGAAGATCTATCTACCTCGGCCCTTGTTTCTTGA	*Bgl*II site (underlined)
dwCre-F	GCCTGCTGGAAGATGGCGATTAG	-
lipTT66-R1	GTGTGAGATACCGTTCGTATAATGTATGCTATACGAAGTTATCTCCACCTGTGTCAATC	-
66upAxp-F	CATTATACGAACGGTATCTCACACCCACCTCCAAG	-
upAxp-R	GGGAGAGCTCTAGAGTCGACACGCGTCGACTAGTCTTTAGAATTGGAAGCTGATC	*Spe*I site (underlined)
hp4d-F	CTAGTCGACGCGTGTCGACTCTAGAGCTC	-
Xpr2pre-R	GGCCAGAACGGCCGTGAGAATAGTAAAG	-
ROL-F	CTATTCTCACGGCCGTTCTGGCCGTGCCTGTGTCTGGCAAGTC	-
kpnROL-R	GAGGTACCCTACAGACAGGAGCCCTCGTTG	*Kpn*I site (underlined)
XprTT-F	CAACGAGGGCTCCTGTCTGTAGGGTACCTCCATGGCCTGTCCCCACGTTG	-
Xprlox71-R	CACGAGGCCCTTTCGTCTTCAAGAATTCGAGACCTCATGTTTGACAGCTTATCATAAC	*Eco*RI site (underlined)
Yvgb-F	CCACAAAGCTTGCCACCATGTTGGATCAACAGACCATTAAC	*Hind*III site (underlined)
Yvgb-R	CAACGTGGGGACAGGCCATGGAGCGGCCGCTTATTCAACAGCTTGAGCG	*Not*I site (underlined)
mLeu-F	GAAGATCTACTGATCACGGGCAAAAG	*Bgl*II site (underlined)

mLeu-R	CATGGTGGCAAGCTTTGTGG	*Hind*III site (underlined)
XprTT-F2	TCCATGGCCTGTCCCCACGTTG	-
Ampup-R	GAAGCATTTATCAGGGTTATTG	-
lipTT66-R2	CTGTCTACCGTTCGTATAATGTATGCTATACGAAGTTATCTCCACCTGTGTCAATC	-
66upXpr-F	CATTATACGAACGGTAGACAGTTAGAGCAGCAACGCG	-
upXpr-R	CTCTAGAGTCGACACGCGTCGACTAGTGGGCCCGTCGATGGGGTTTATATC	*Spe*I site (underlined)
pENO$_{100}$-F3	GGAAGATCTAGCGCAATTGGGTTTGCATCATGTA	*Bgl*II site (underlined)
pENO$_{100}$-F4	CGCGGATCCAGCGCAATTGGGTTTGCATCATGTA	*Bam*HI site (underlined)
pENO-R2	CATGGTGGCAAGCTTGGTGATAAATGTGTGGTTAGACG	*Hind*III site (underlined)
pENO-R3	ATTTGCGGCCGCGGTGATAAATGTGTGGTTAGACG	*Not*I site (underlined)
Kar2-F	ATCACCAAGCTTGCCACCATGAAGTTCTCTATGCCTTCGTGGG	*Hind*III site (underlined)
Kar2-R	AAGGAAAAAAGCGGCCGCTTAAAGCTCATCGTGGAAAGGAGCC	*Not*I site (underlined)
Pdi-F	ATCACCAAGCTTGCCACCATGAAGTTCACTGCCCTCACAATTG	*Hind*III site (underlined)
Pdi-R	AAGGAAAAAAGCGGCCGCTTAAAGCTCATCATCAATCTTGCCT	*Not*I site (underlined)
Hac1-F	ATCACCAAGCTTGCCACCATGTCTATCAAGCGAGAAGAGTCCT	*Hind*III site (underlined)
Hac1-R	AAGGAAAAAAGCGGCCGCTCACTCCAATCCCCCAAACAGGTTG	*Not*I site (underlined)
Axpout-R	CGACCTGGAGAAGATCTGTG	-
qact1-F	CTGGCCGAGATCTTACCGAC	RT-PCR for *act1* gene
qact1-R	CATCGGGAAGCTCGTAGGAC	RT-PCR for *act1* gene

qtll-F	CTGGACAACACCAACAAGCTG	RT-PCR for *tll* gene
qtll-R	GTCCTCAACCTTCTGTCGGAG	RT-PCR for *tll* gene

Supplementary Figures

Figure captions

Figure S1. PCR identification of several Po1f/tll strains. (A) PCR using primers XprTT-F2 and Axpout-R, CK: Po1f (control); (B) PCR using primers TLL-F and TLL-R, CK: pUAxp7166-hp16e_{100}-tll (control); H_2O: water (control).

Figure S2. (A) Effect of glycosylation on TLL expressed by *Y. lipolytica*; lane M, molecular weight marker; lane 1, Po1f/tll fermentation supernatant; lane 2, deglycosylation of TLL expressed by Po1f/tll; lane 3, Endo H; (B) Mass spectrometry results for TLL, partially matched peptides are shown in red.

Figure S3. Optimization of TLL fermentation in 500-mL shake flasks. (A) Effects of different D-sorbitol concentrations on TLL production; (B) Relationship between TLL activity and inoculation density; (C) Influences of different initial pH on TLL production; (D) Effects of culture medium volume on TLL expression. (E) Time course of TLL expression.

Figure S4. Schematic diagram of recombinant plasmids. (A) hp4d-egfp; (B) pENOn-egfp (n = 75, 100, 125, 150, 175, 200, 300, 400, 700, 1000, and 1300 bp); (C) hp4d-tll; (D) pENOn-tll (n = 100, 125, 200, 400, 700, 1000, and 1300 bp); (E) hp4e_n-tll (n = 100, 125, 200, 400, 700, 1000, and 1300 bp).

Figure S5. Flow diagram of the steps used for construction of expression vectors hpxe$_{100}$-tll (x = 8, 12, 16, 20, 24, 28, and 32) harboring different copies of UAS1B.

Figure S6. Schematic diagram of recombinant plasmids. (A) pUAxp7166-hp16e$_{100}$-tll; (B) pUXpr7166-type plasmids, helper gene means hp4e$_{100}$ (control), *hac1*, *kar2*, and *pdi*, respectively.

Figure S1

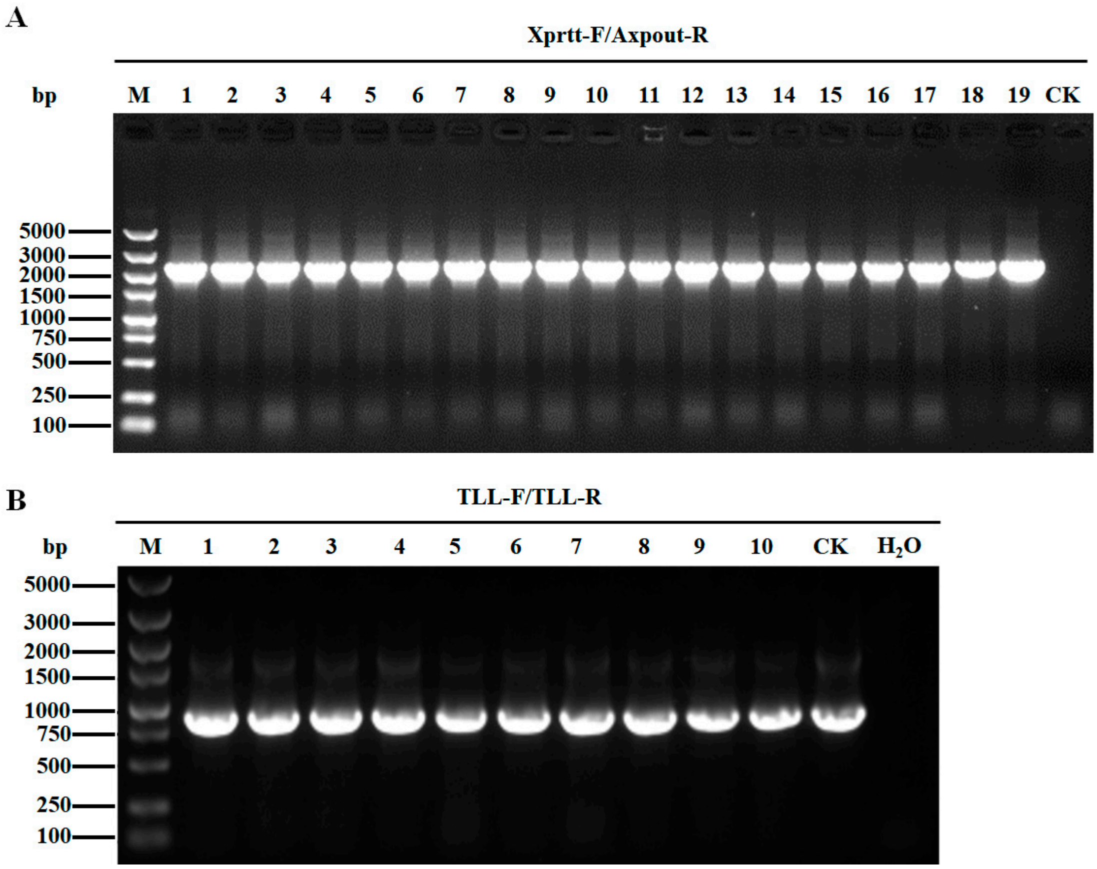

A
Xprtt-F/Axpout-R
bp
M 1 2 3 4 5 6 7 8 9 10 11 12 13 14 15 16 17 18 19 CK
5000
3000
2000
1500
1000
750
500
250
100
B
TLL-F/TLL-R
bp
M 1 2 3 4 5 6 7 8 9 10 CK H2O
5000
3000
2000
1500
1000
750
500
250
100

Figure S2

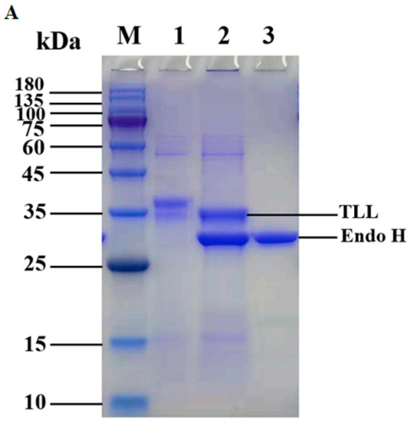

B

1 MRSSLVLFFVSAWTALASPIRREVSQDLFNQFNLFAQYSAAAYCGKNNDA

51 PAGTNITCTGNACPEVEKADATFLYSFEDSGVGDVTGFLALDNTNKLIVL

101 SFRGSRSIENWIGNLNFDLKEINDICSGCRGHDGFTSSWRSVADTLRQKV

151 EDAVREHPDYRVVFTGHSLGGALATVAGADLRGNGYDIDVFSYGAPRVGN

201 RAFAEFLTVQTGGTLYRITHTNDIVPRLPPREFGYSHSSPEYWIKSGTLV

251 PVTRNDIVKIEGIDATGGNNQPNIPDIPAHLWYFGLIGTCL*

Figure S3

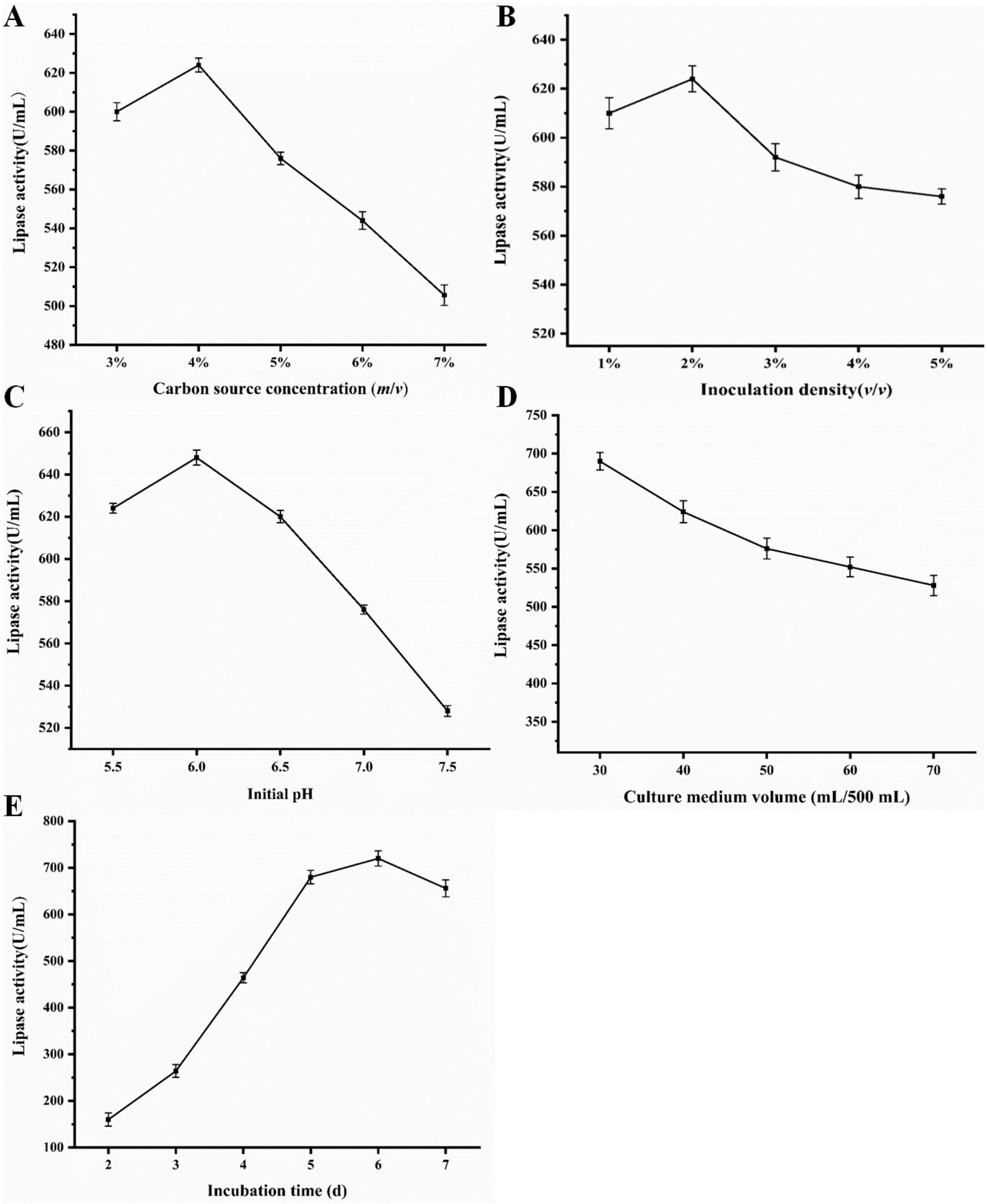
A
Lipase activity(U/mL)
640
620
600
580
560
540
520
500
480
3%
4%
5%
6%
7%
Carbon source concentration (*m*/*v*)
B
Lipase activity(U/mL)
640
620
600
580
560
540
520
1%
2%
3%
4%
5%
Inoculation density(*v*/*v*)
C
Lipase activity(U/mL)
660
640
620
600
580
560
540
520
5.5
6.0
6.5
7.0
7.5
Initial pH
D
Lipase activity(U/mL)
750
700
650
600
550
500
450
400
350
30
40
50
60
70
Culture medium volume (mL/500 mL)
E
Lipase activity(U/mL)
800
700
600
500
400
300
200
100
2
3
4
5
6
7
Incubation time (d)

Figure S4

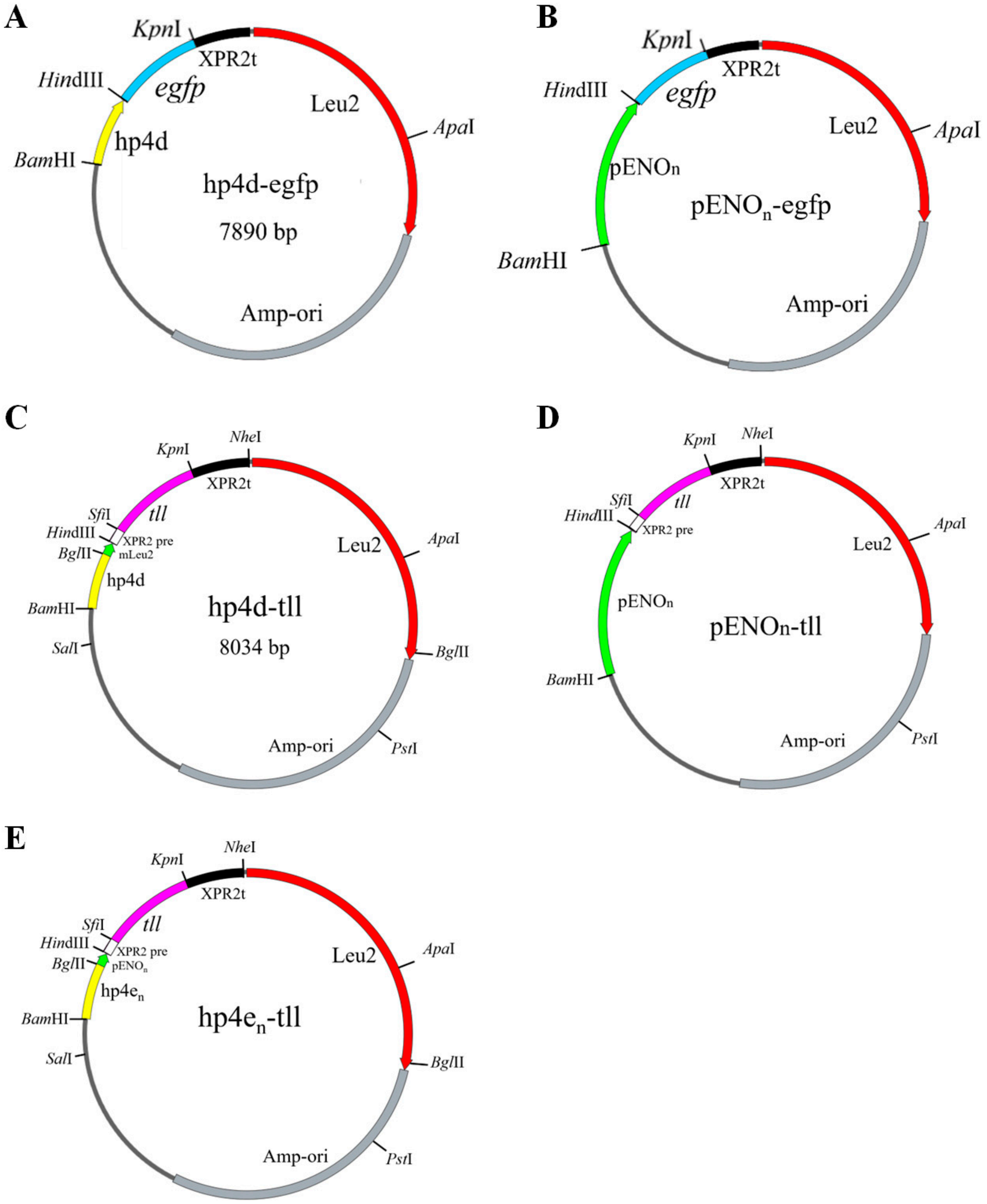
A
KpnI
XPR2t
HindIII
egfp
Leu2
ApaI
hp4d
BamHI
hp4d-egfp
7890 bp
Amp-ori
B
KpnI
XPR2t
HindIII
egfp
Leu2
ApaI
pENOn
pENOn-egfp
BamHI
Amp-ori
C
NheI
KpnI
XPR2t
SfiI
tll
HindIII
XPR2 pre
BglII
mLeu2
Leu2
ApaI
hp4d
BamHI
hp4d-tll
SalI
8034 bp
BglII
Amp-ori
PstI
D
NheI
KpnI
XPR2t
SfiI
tll
HindIII
XPR2 pre
ApaI
Leu2
pENOn
pENOn-tll
BamHI
Amp-ori
PstI
E
KpnI
NheI
XPR2t
SfiI
tll
HindIII
XPR2 pre
BglII
pENOn
Leu2
ApaI
hp4en
BamHI
hp4en-tll
SalI
BglII
Amp-ori
PstI

Figure S5

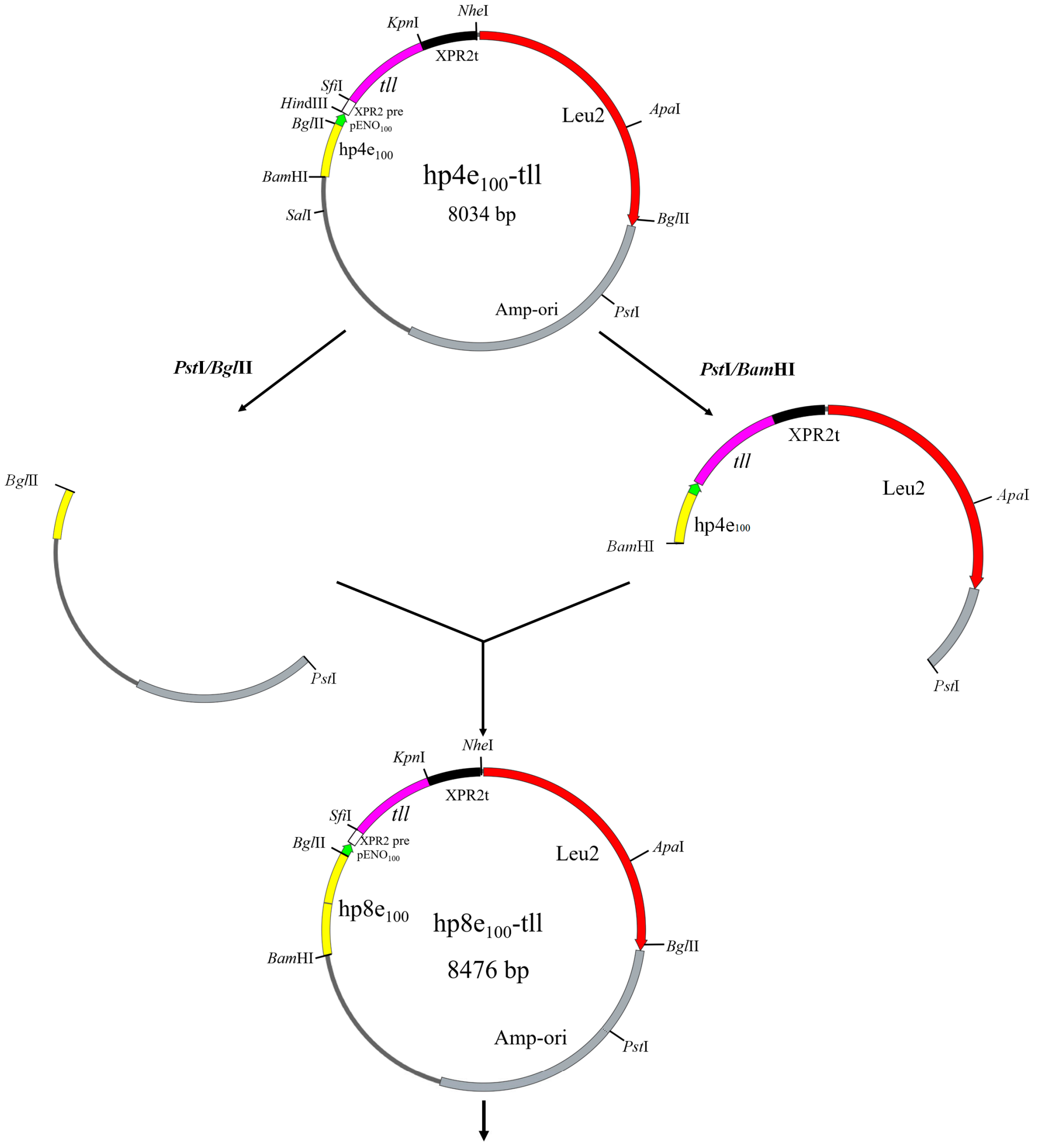

Repeat the above steps to construct hpxe$_{100}$-tll
(x = 12, 16, 20, 24, 28, and 32)

Figure S6

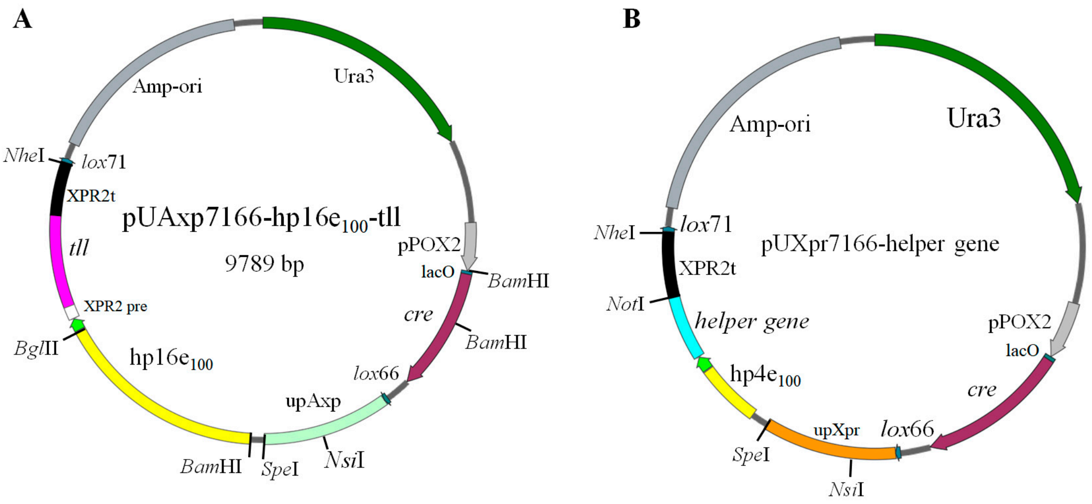
A
Amp-ori
Ura3
NheI
lox71
XPR2t
pUAxp7166-hp16e100-tll
tll
9789 bp
pPOX2
lacO
BamHI
cre
BamHI
XPR2 pre
BglII
hp16e100
lox66
upAxp
BamHI
SpeI
NsiI
B
Amp-ori
Ura3
NheI
lox71
pUXpr7166-helper gene
XPR2t
NotI
helper gene
pPOX2
lacO
hp4e100
cre
upXpr
lox66
SpeI
NsiI
